# Myoglobin for Detection of High-Risk Patients with Acute Myocarditis

**DOI:** 10.1007/s12265-020-09957-8

**Published:** 2020-01-31

**Authors:** Jan Kottwitz, Katelyn A. Bruno, Jan Berg, Gary R. Salomon, DeLisa Fairweather, Mawahib Elhassan, Nora Baltensperger, Christine K. Kissel, Marina Lovrinovic, Andrea Baltensweiler, Christian Schmied, Christian Templin, Joao A.C. Lima, Ulf Landmesser, Thomas F. Lüscher, Robert Manka, Bettina Heidecker

**Affiliations:** 1 Emergency Department, University Hospital of Zurich, Zürich, Switzerland; 2 Department of Anesthesiology, Intensive Care, Rescue and Pain Medicine, Kantonsspital St. Gallen, St. Gallen, Switzerland; 3 Department of Cardiovascular Medicine, Mayo Clinic, Jacksonville, FL USA; 4 Department of Cardiology, University Hospital of Zurich, Zürich, Switzerland; 5 Department of Cardiology, The Johns Hopkins University, Baltimore, MD USA; 6 Department of Cardiology, Charite Universitätsmedizin Berlin, Campus Benjamin Franklin, Hindenburgdamm 30, 12203 Berlin, Germany; 7 Center for Molecular Cardiology, University of Zurich, Zürich, Switzerland; 8 Royal Brompton and Harefield Hospitals and Imperial College, London, UK

**Keywords:** Magnetic resonance imaging, Late gadolinium enhancement, Myocarditis, Myocardial inflammation, Cardiac enzymes, Myoglobin, Troponin, Biomarker

## Abstract

There is an unmet need for accurate and practical screening to detect myocarditis. We sought to test the hypothesis that the extent of acute myocarditis, measured by late gadolinium enhancement (LGE) on cardiac magnetic resonance imaging (CMR), can be estimated based on routine blood markers. A total of 44 patients were diagnosed with acute myocarditis and included in this study. There was strong correlation between myoglobin and LGE (r_s_ = 0.73 [95% CI 0.51; 0.87], *p* < 0.001), while correlation was weak between LGE and TnT-hs (r_s_ = 0.37 [95% CI 0.09; 0.61], *p* = 0.01). Receiver operating curve (ROC) analysis determined myoglobin ≥ 87 μg/L as cutoff to identify myocarditis (92% sensitivity, 80% specificity). The data were reproduced in an established model of coxsackievirus B3 myocarditis in mice (*n* = 26). These data suggest that myoglobin is an accurate marker of acute myocarditis.

**Electronic supplementary material:**

The online version of this article (10.1007/s12265-020-09957-8) contains supplementary material, which is available to authorized users.

**Graphical Abstract:**
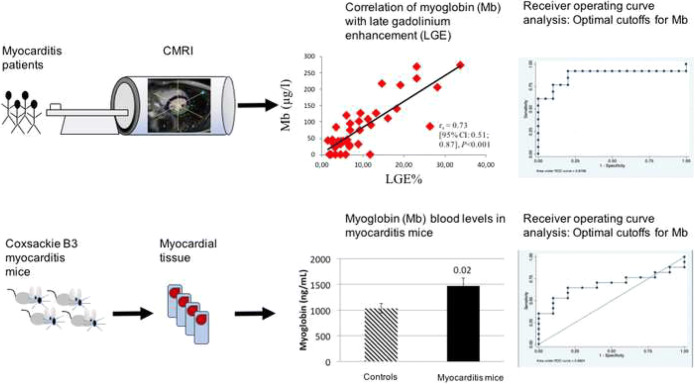
Receiver operating curve analysis determined myoglobin ≥ 87 μg/L as cutoff to identify myocarditis and these data were reproduced in an established model of coxsackievirus B3 myocarditis in mice: CMRI, cardiac magnetic resonance imaging; Mb, myoglobin; LGE, late gadolinium enhancement; ROC, receiver operating curve analysis.

## Introduction

Myocarditis is a common inflammatory cardiomyopathy presenting with various clinical manifestations and degrees of severity [1–3]. It remains one of the most important causes of dilated cardiomyopathy (DCM) with up to 30% of cases progressing to DCM [4]. Furthermore, myocarditis is one of the most common causes of sudden cardiac death in young adults [5]. Its etiology is often viral or autoimmune [1, 3]. As of today, accurate diagnosis of myocarditis and identification of individuals at highest risk for adverse cardiovascular events remain a challenge [6]. While endomyocardial biopsy (EMB) is the gold standard for definitive diagnosis of myocarditis [1], the risk of complications and potential sampling error led to its use primarily in a selected group of patients as outlined per AHA/ACC guidelines [2, 7, 8]. Cardiac magnetic resonance imaging (CMR) is a valuable non-invasive alternative if EMB is not indicated [9–15]. Furthermore, late gadolinium enhancement (LGE) on CMR has been shown to predict poor outcomes [15–17]. Two recent studies reported that the localization of LGE predicts the degree of severity of adverse cardiovascular outcomes [17, 18]. Thus, there is a major need for accurate and practical diagnostic screening tests to identify high-risk patients with acute myocarditis and refer them to CMR for assessment of LGE. Circulating cardiac and inflammatory markers are routinely obtained from patients with acute myocarditis with TnT-hs being the gold standard [1, 2, 19]. However, it is uncertain if these markers reflect disease severity [1, 3, 6, 20–22]. In this study, we sought to test the hypothesis that the extent of acute myocarditis, measured by LGE on CMR, can be estimated based on routine cardiac and inflammatory markers.

## Methods

### Patient Population

This is a single-center retrospective chart review of all patients who presented to the University Hospital of Zurich with suspected acute myocarditis from January 2011 through December 2017. The local Ethics Committee of the Kanton of Zurich approved the study protocol.

The patient population consisted of a myocarditis group and a control group:

1. Myocarditis cohort

This cohort included patients diagnosed with myocarditis from January 2011 through March 2017. Myocarditis was diagnosed based on clinical presentation, elevation of TnT-hs [1, 2] and CMR, after exclusion of obstructive coronary artery disease through coronary angiography. Coronary angiography was primarily performed by cardiac catheterization or computed tomography if pretest probability for coronary artery disease was very low. Forty-four out of eighty-four myocarditis patients diagnosed with acute myocarditis met inclusion criteria for the study. To create a homogenous cohort that only reflects acute myocarditis, the analysis was restricted to patients with recent symptom onset (≤ 10 days) and patients with myositis were excluded from the study.

Symptom onset was defined by the occurrence of at least one of the following complaints: chest pain, dyspnea, new onset or worsening heart failure, severe arrhythmias, syncope or cardiac arrest. The primary cohort served to test the correlation of cardiac and inflammatory markers with LGE. Subsequently, receiver operating curve (ROC) analysis was performed to identify if the marker with the strongest correlation to LGE can be used for diagnostic screening to identify patients with myocarditis and to test its diagnostic accuracy.

2. Control group

The second cohort consisted of 22 patients with similar signs and symptoms as the primary cohort, including elevation of TnT-hs. Patients with obstructive coronary artery disease were not included in the control group, and myocarditis was not supported by CMR. Therefore, they were classified as myocardial infarction with non-obstructive coronary arteries (MINOCA). The control group was primarily used to test the accuracy of the biomarker in distinguishing patients with myocarditis and LGE on CMR from MINOCA.

#### Patients Excluded

A total of 15 patients had missing data and one patient withdrew consent. Twenty-four patients were excluded due to chronic or recurrent myocarditis, symptoms > 10 days, and/or known myositis.

### Clinical Laboratory Parameters

Blood parameters were analyzed on the day of admission: high-sensitivity troponin T (TnT-hs), creatine kinase (CK), myoglobin, NT-pro brain natriuretic peptide (NT-proBNP), C-reactive protein (CRP), leukocytes and thrombocytes. Values for TnT-hs, CK, myoglobin, and CRP were obtained 3, 6, 12, 24, 48, and 72 h after admission.

### CMR Examination

CMR imaging was obtained within 10 days after symptom onset. Images of CMR were performed on a 1.5- or 3.0-Tesla scanner (SiemensSkyra, Erlangen, Germany or Philips Achieva, Best, The Netherlands) using an electrocardiography-gated breath-hold protocol. Diagnosis was based on cine-CMR, T2-weighted imaging, and T1-weighted LGE imaging. For details on CMR examination, please see supplement.

### Clinical Follow-Up

Patients presented for 3 and 6 months follow-up, at which time they were assessed for major adverse cardiovascular events (MACE) such as malignant arrhythmias, severe chest pain or dyspnea episodes, new onset heart failure, cardiovascular death, and all cause-mortality. During physical examination, the set of circulating cardiac and inflammatory markers were obtained as outlined above. All patients underwent 12-lead ECG, 48-h Holter monitoring, and exercise stress testing with ramp protocol.

### Validation of Findings in a Clinically Translatable Mouse Model of Coxsackievirus B3 Induced Myocarditis

Male and female BALB/c (6 to 8 weeks old) mice were obtained from the Jackson Laboratory (Bar Harbor, ME) fed standard chow and housed with corn cobb bedding in animal rooms where temperature was monitored. Mice were maintained under pathogen-free conditions in the animal facility at Mayo Clinic. Approval was obtained from the Animal Care and Use Committee of Mayo Clinic for all procedures. Mice were inoculated intraperitoneally (ip) with 10^3^ plaque forming units of a heart-passaged stock of CVB3 diluted in saline or saline alone (controls) on day 0 and myocarditis examined at day 10 post infection (pi), according to prior studies [23]. Knowledge of which mice were infected with CVB3 vs. saline controls were necessary so that samples could be handled safely by the researcher because CVB3 is a BSL2 pathogen. Mice were randomly assigned to groups using simple randomization. Myocarditis was assessed histologically as the percentage of the heart section with inflammation compared to the overall size of the heart section, as previously (Fairweather 2014, Coronado 2019). Analysis of samples was blinded to the researcher using a code. No deaths of mice occurred in any of the experiments reported in this manuscript. Sera samples, collected on day 10pi, from individual uninfected or CVB3 exposed male and female mice were used to measure myoglobin (catalog no. MYO-1) and ultrasensitive troponin (catalog no. CTNI-1-US) using Life Diagnostic Inc. ELISA kits (West Chester, PA) according to the manufacturer’s instructions and expressed as ng/mL.

### Statistical Analysis

Clinical data are reported as mean ± standard deviation or median interquartile range (IQR). Animal data are expressed as mean ± standard error of the mean or as Pearson correlation coefficient (r_p_). Correlation between clinical laboratory parameters and percentage of LGE of left ventricular myocardial volume on CMR imaging was analyzed using Spearman rank correlation coefficient (r_s_). *p* < 0.05 was considered statistically significant based on a two-tailed probability. Patients with myocarditis and LGE > 10% were included in the primary cohort, in which correlation analysis and development of the biomarker was performed [17]. Please find details on statistical analysis in the supplement.

## Results

### Patient Characteristics

Forty-four patients diagnosed with acute myocarditis during the years 2011–2017 met inclusion criteria for the study. Twenty-two patients with myocardial infarction with non-obstructive coronary arteries (MINOCA), in whom CMR excluded myocarditis, served as the control group.

Among patients with acute myocarditis, 82% were men with a mean age of 42 ± 17 years. Twenty-five percent were smokers, 11% suffered from concomitant coronary artery disease, 20% from arterial hypertension, and 5% from type II diabetes. Baseline LVEF was 54 ± 12% based on CMR data. The mean body mass index (BMI) in patients with myocarditis was 27 ± 4 kg/m^2^. In the control group, 86% were male with a mean age of 43 ± 16 years and 32% were smokers. Two patients (9%) had concomitant coronary artery disease, 18% arterial hypertension, and 5% were diabetics. The mean BMI of controls was 26 ± 5 kg/m^2^ and baseline LVEF was 53 ± 14% based on CMR.

Baseline characteristics of the primary cohort were overall similar between myocarditis (*n* = 44) and controls (*n* = 22, Table 1). However, TnT-hs and myoglobin levels were higher in patients with myocarditis compared to controls. While the mean TnT-hs was 492 ± 645 ng/L in myocarditis, it was 22 ± 18 ng/L in controls (*p* = 0.03). Mean myoglobin levels were 77 ± 75 μg/L in myocarditis and 24 ± 14 μg/L in controls (*p* = 0.03). Among 44 patients with myocarditis, there were 18 patients in whom both levels of myoglobin as well as TnT-hs were elevated. In patients with elevation of myoglobin and TnT-hs, there was 16 LGE% of left ventricular volume vs 4 LGE % of left ventricular volume in the group without elevation of both makers. In addition, CK levels were elevated in myocarditis compared to controls (290 ± 219 U/L vs 116 ± 74 U/L; *p* = 0.01).

**Table 1:** Baseline characteristics of patients with acute myocarditis vs. controls

Characteristics	Acute Myocarditis (*n* = 44)	Controls (*n* = 22)	*p* value
Mean age, (SD)	42 (17)	43 (16)	0.90
Male sex, *n* (%)	36 (82)	19 (86)	0.74
BMI, mean, (SD)	27 (4)	26 (5)	0.65
Smoker, *n* (%)	11 (25)	7 (32)	0.77
CAD, *n* (%)	5 (11)	2 (9)	1
aHTN, *n* (%)	9 (20)	4 (18)	1
DM, *n* (%)	2 (5)	1 (5)	1
LVEF, %, (SD)	54 (12)	53 (14)	0.76
TnT-hs, ng/L, (SD)	492 (645)	22 (18)	0.03*
CK, U/L, (SD)	290 (219)	116 (74)	0.01*
Mb, μg/L, (SD)	77 (75)	24 (14)	0.03*
NT-proBNP, ng/L, (SD)	1299 (3105)	291 (288)	0.34
CRP, mg/L, (SD)	30 (38)	13 (20)	0.14
Lc, G/L, (SD)	8 (3)	7 (3)	0.09
Tc, G/L, (SD)	247 (86)	231 (88)	0.59

*aHTN* arterial hypertension, *BMI* body mass index, *CAD* coronary artery disease, *CK* creatine kinase, *CRP* C-reactive protein, *DM* diabetes mellitus, *Lc* leukocytes, *LVEF* left ventricular ejection fraction, *Mb* myoglobin, *NT-pro BNP* NT-pro brain natriuretic peptide, *SD* standard deviation, *Tc* thrombocytes, *TnT-hs* high sensitivity troponin T

*p value < 0.05

At presentation, 36 patients (82%) complained of chest pain, whereas dyspnea was the leading symptom in eight patients (18%). Additional symptoms were acute decompensated heart failure in three (7%), palpitations in four (9%), and syncope in two patients (5%). ECG revealed ST segment elevation in 12 (27%) and ST segment depression in four patients (9%).

### Reproducibility of CMR Interpretation

As previously described by our group [24], intrareader reproducibility for interpretation of CMR data was excellent (r^2^ = 0.97 in linear regression; *p* = 0.59 in Bland-Altman). There was also a strong correlation between CMR interpretation results between the two readers (r^2^ = 0.99). In addition, no proportional bias (*p* = 0.82) was observed between the two readers based on Bland Altman analysis.

### Correlation Between Laboratory Blood Parameters and LGE on CMR in Acute Myocarditis

**Fig. 1:**
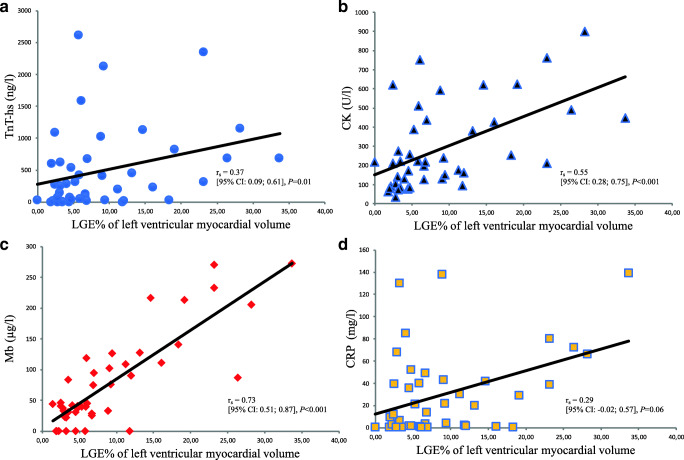
Correlation of circulating cardiac and inflammatory markers with late gadolinium enhancement (LGE) in acute myocarditis: LGE as % of left ventricular myocardial volume is illustrated on the x-axis. Levels of **a** high-sensitivity troponin T (TnT-hs), **b** creatine kinase (CK), **c** myoglobin (Mb), and **d** C-reactive protein (CRP) are demonstrated on the y-axis. r_s_ = Spearman rank correlation coefficient

CMR imaging data for individual patients are illustrated in the supplement (Table S1). Median values and ranges of the analyzed laboratory parameters for the entire primary cohort are displayed in Table 2. Strong and significant correlation was found between myoglobin and LGE (r_s_ = 0.73 [95% CI 0.51; 0.87], *p* < 0.001, Fig. 1). There were weak to moderate correlations between LGE and CK (r_s_ = 0.55 [95% CI 0.28; 0.75], *p* < 0.001), TnT-hs (r_s_ = 0.37 [95% CI 0.09; 0.61], *p* = 0.01), and CRP (r_s_ = 0.29 [95% CI -0.02; 0.57], *p* = 0.06) (Fig. 1). There was also a weak correlation between LGE and NT-proBNP (r_s_ = 0.34 [95% CI 0.03; 0.59], *p* = 0.04) and Lc (r_s_ = 0.23 [95% CI − 0.05; 0.49], *p* = 0.13), and no correlation was found between LGE and Tc (r_s_ = 0.12 [95% CI − 0.18; 0.39], *p* = 0.46). Mean baseline levels of myoglobin at symptom onset were 77 ± 75 μg/L in myocarditis vs. 24 ± 14 μg/L in controls (*p* = 0.03). Additional baseline parameters are displayed in Table 1.

**Table 2:** Correlation between laboratory markers and LGE in acute myocarditis (*n* = 44)

Laboratory marker	Reference range in laboratory	Median (IQR) at baseline	Correlation with LGE at baseline
TnT-hs (ng/L)	< 14	267 (31–682)	r_s_ = 0.37 [95% CI: 0.09; 0.61], *p* = 0.01
CK (U/L)	< 190	220 (129–436)	r_s_ = 0.55 [95% CI: 0.28; 0.75], *p* < 0.001
Mb (μg/L)	28–72	43 (30–111)	r_s_ = 0.73 [95% CI: 0.51; 0.87], *p* < 0.001
NT-pro BNP (ng/L)	< 85.5	441 (70–951)	r_s_ = 0.34 [95% CI: 0.03; 0.59], *p* = 0.04
CRP (mg/L)	< 5	14 (2–43)	r_s_ = 0.29 [95% CI: −0.02; 0.57], *p* = 0.06
Lc (G/L)	3–9.6	8 (6–10)	r_s_ = 0.23 [95% CI: −0.05; 0.49], *p* = 0.13
Tc (G/L)	150–360	223 (196–267)	r_s_ = 0.12 [95% CI: −0.18; 0.39], *p* = 0.46

*CK* creatine kinase, *CRP* C-reactive protein, *IQR* interquartile range, *Lc* leukocytes, *LGE* late gadolinium enhancement, *Mb* myoglobin, *NT-pro BNP* NT-pro brain natriuretic peptide, *Tc* thrombocytes, *TnT-hs* high sensitivity troponin T

Furthermore, we sought to evaluate if myoglobin and LGE correlated with LVEF, since it appeared plausible that an increased extent of LGE would affect left ventricular systolic function. Correlations between myoglobin and LVEF (r_s_ = − 0.30 [95% CI − 0.55; 0.01], *p* = 0.05), as well as LGE and LVEF (r_s_ = − 0.29 [95% CI -0.56; 0.01], *p* = 0.06) were weak and did not reach statistical significance.

### Myoglobin and Troponin as Predictors of LGE on CMR in Acute Myocarditis

**Fig. 2:**
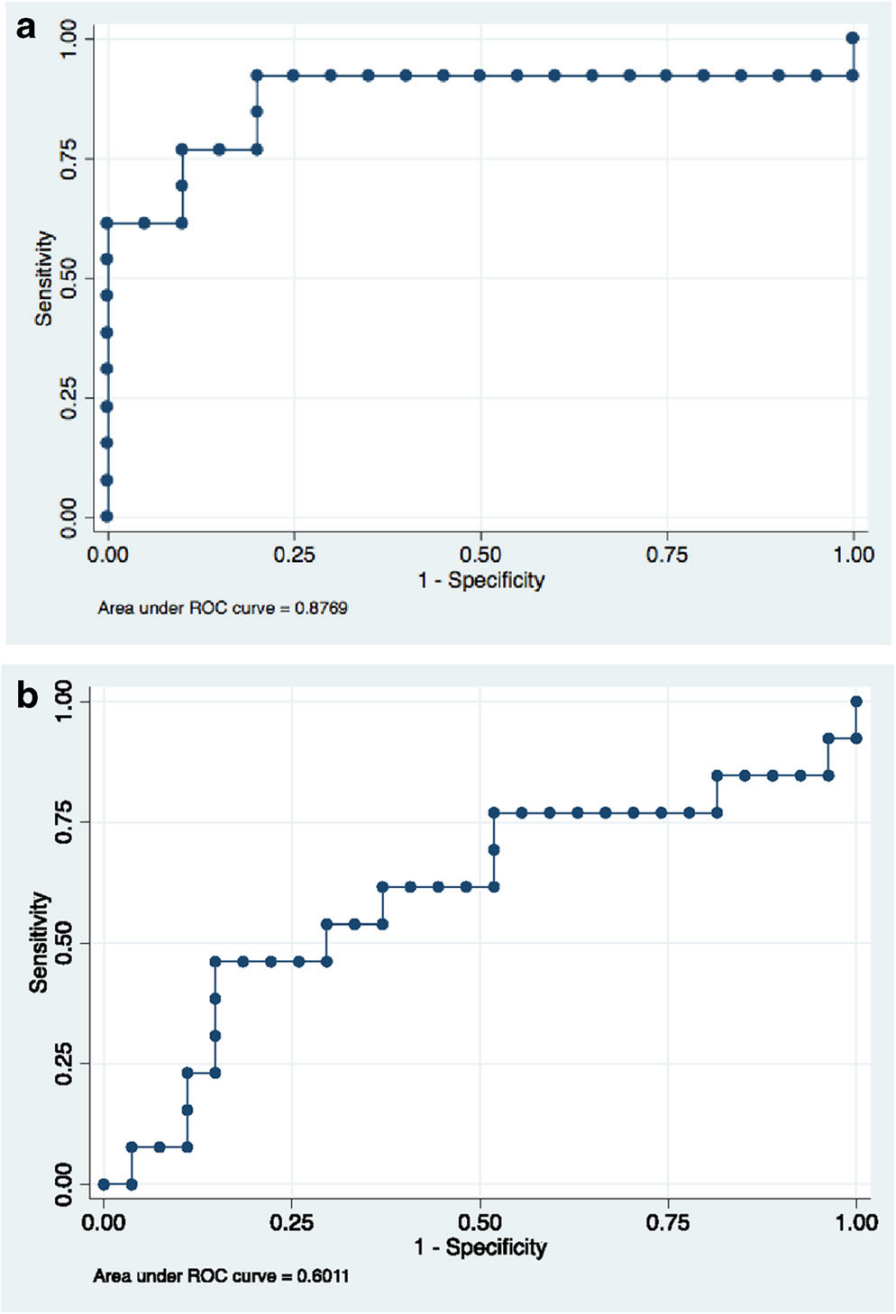
Receiver operating curve analysis to obtain optimal cutoffs for myoglobin (panel **a**) and high sensitivity troponin T (panel **b**) to identify patients with acute myocarditis and LGE on CMR

As we found a strong correlation between myoglobin and LGE, we sought to determine the optimal cutoff for serum myoglobin to detect acute myocarditis on CMR. ROC analysis revealed myoglobin ≥ 87 μg/L to be the optimal cut off (area under the curve, AUC = 0.88 [95% CI 0.72; 0.97], Fig. 2a) with 92% sensitivity, 80% specificity, and 85% accuracy.

We also performed ROC analysis for TnT-hs, the laboratory “gold standard method” to detect myocarditis clinically, in order to test if an optimized cutoff could be developed and to compare it to the standard reference range that is currently used in clinical practice. For TnT-hs, an optimized cutoff value of 201 ng/L was determined through ROC analysis, which resulted in 77% sensitivity, 48% specificity, and AUC = 0.60 (Fig. 2b). In addition, we tested the diagnostic accuracy of the clinical reference range of TnT-hs [0–14 ng/L] which resulted in 100% sensitivity, 22% specificity, and 59% accuracy.

### Time Course of Laboratory Blood Parameters

**Fig. 3:**
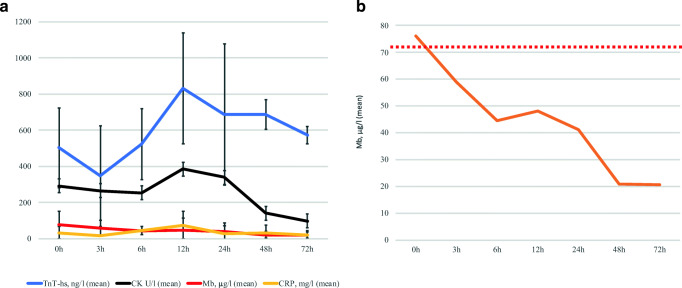
Time course of cardiac and inflammatory markers during acute myocarditis: Time points of blood collection are illustrated on the x-axis. Mean levels of the examined blood parameters are demonstrated on the y-axis. Standard deviation is indicated in panel **a**. Panel **b** shows mean levels of myoglobin at given time points. The dotted red line indicates the upper reference value of myoglobin. CK, creatine kinase; CRP, C-reactive protein; Mb, myoglobin; TnT-hs, high sensitivity troponin T

The time course of cardiac markers and CRP after admission with myocarditis is illustrated in Fig. 3a. Mean TnT-hs peaked at 12 h (mean: 831 ± 822 ng/L) and continued to be elevated for at least 72 h. Mean CK peaked at 12 h (mean 384 ± 306 U/L) and normalized 48 h post admission. Mean myoglobin levels peaked on admission (mean 77 ± 75 μg/L) and normalized on average 3 h later (Fig. 3b). Twenty-four hours post admission, myoglobin levels had normalized in all patients. Mean CRP peaked after 12 h (75 ± 77 mg/L) and was still detectable 72 h later (mean 20 ± 27 mg/L).

### Major Adverse Cardiovascular Events in Patients with Acute Myocarditis

MACE occurred in four patients over a period of 6 months. At 3 months follow-up, two patients with myocarditis underwent implantation of an intracardiac device. One patient underwent implantation of a cardioverter defibrillator (ICD) for ventricular fibrillation. Another patient received an implantable cardiac resynchronization therapy pacemaker (CRT-P) for persistent symptomatic second-degree atrioventricular block. At 6 months follow-up, two patients underwent insertion of an ICD for symptomatic ventricular tachycardia (VT) with syncope. Among the four patients, three were men (75%). The average age in men was 43 ± 14 years, while the woman was 43 years old. Myoglobin levels were elevated above the reference value of 72 μg/L at baseline in three of the four patients with MACE (myoglobin mean level 124 ± 106 μg/L), while the mean was 73 ± 71 μg/L in patients without MACE (*p* = 0.20). Three out of the four patients with MACE had elevated levels of both myoglobin and TnT-hs. No MACE were observed in the control group, who had a mean myoglobin level of 24 ± 14 μg/L.

### Reproducibility of Results in a Clinically Translational Model of Coxsackievirus B3 Myocarditis

Since patients in our cohort were diagnosed with myocarditis based on clinical presentation (i.e., elevation of TnT-hs [1, 2] and CMR after exclusion of obstructive coronary artery disease through coronary angiography, without a definitive tissue diagnosis), we sought to validate our findings in a clinically translational mouse model of coxsackievirus B3 (CVB3) myocarditis that closely resembles the time-course and pathogenesis of myocarditis is humans [25, 26]. After intraperitoneal injection with heart-passaged CVB3 [23, 27], male and female mice developed acute myocarditis at day 10 post infection. Myocarditis was confirmed based on histology after the mice developed myocarditis. Ten mice received a saline control injection, were not infected, and served as controls.

**Fig. 4:**
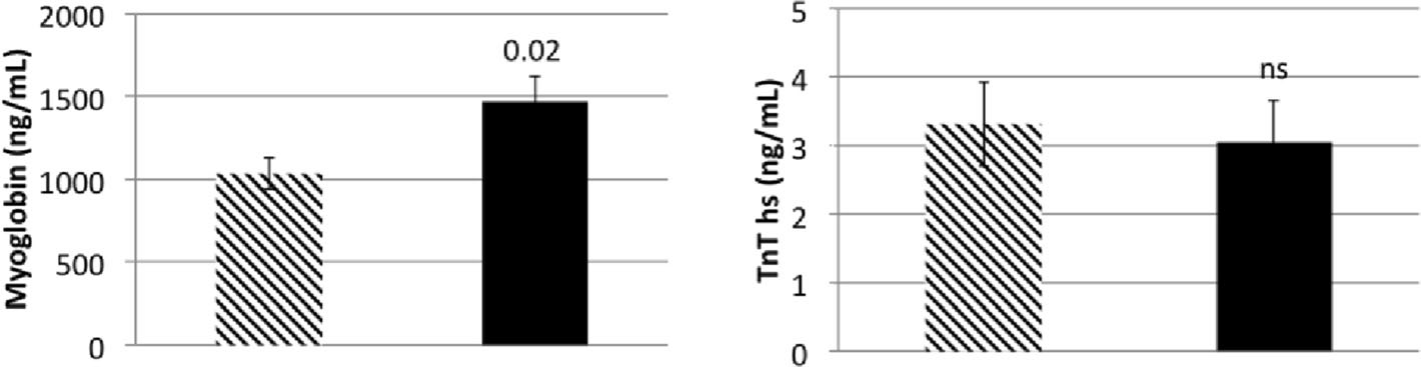
Myoglobin and high sensitivity troponin T (TnT-hs) blood levels in mice infected with heart-passaged CVB3 vs. saline controls. The panel on the left depicts myoglobin blood levels of controls (bar with diagonal stripes, *n* = 10 mice) and of mice with myocarditis during acute myocarditis at day 10 post infection (bar in solid black, *n* = 16 mice). Myoglobin was significantly elevated in myocarditis (*p* = 0.02). The panel on the right depicts blood levels of TnT-hs of controls (bar with diagonal stripes) and of mice with myocarditis (bar in solid black). There was no significant difference in TnT-hs blood levels between mice with myocarditis vs. controls. Data show the mean ± SEM. Significance determined using Student’s *t* test

**Fig. 5:**
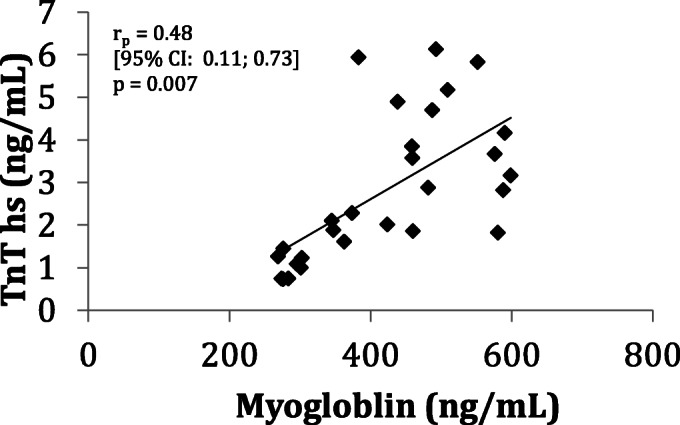
Correlation between high sensitivity troponin T and myoglobin in sera from mice with CVB3 myocarditis at day 10 post infection (r_p_ = 0.48 R^2^ = 0.41, *p* = 0.007). r_p_ = Pearson’s correlation coefficient. *n* = 26 mice

**Fig. 6:**
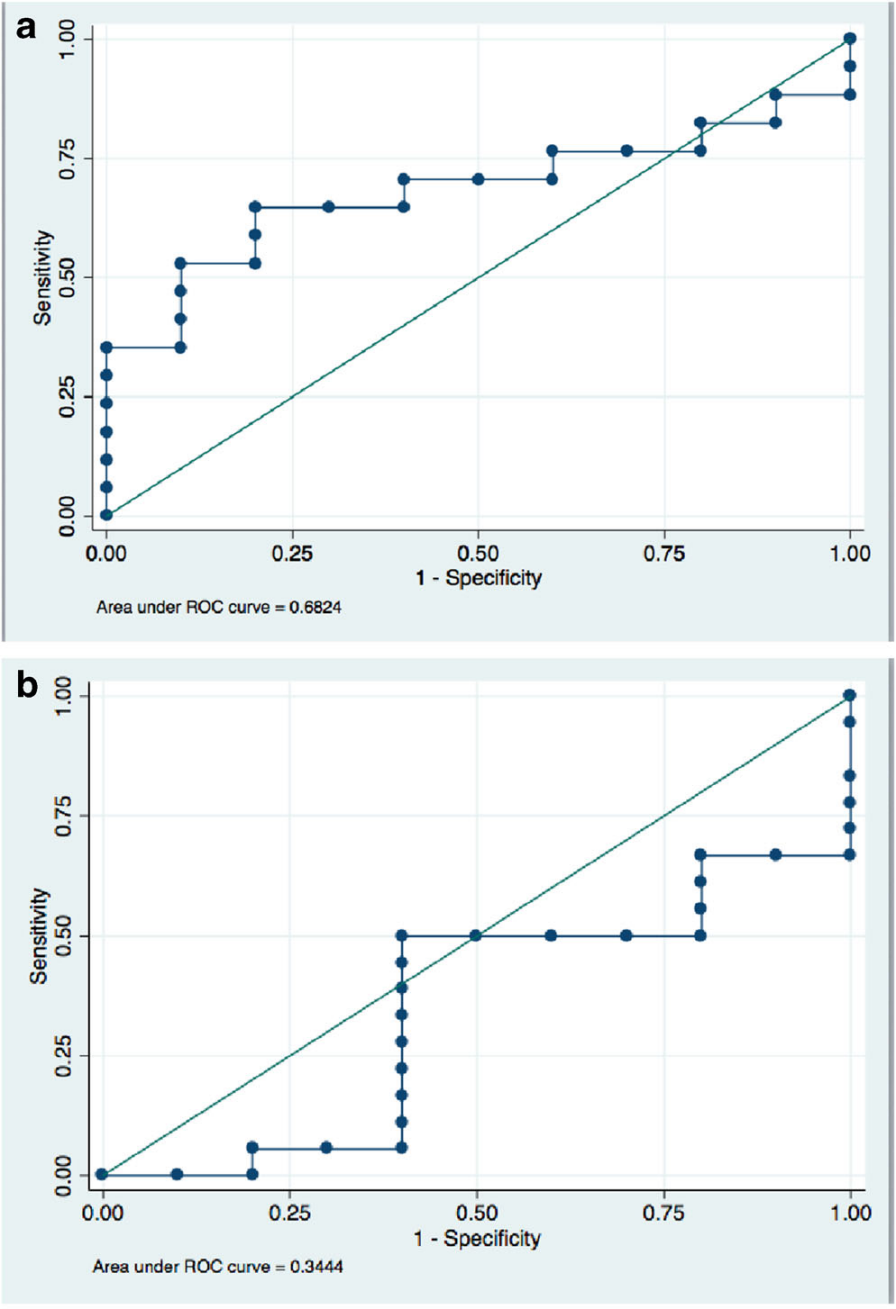
Receiver operating curve analysis to obtain optimal cutoffs for myoglobin (panel **a**) and high sensitivity troponin T (panel **b**) to assess diagnostic accuracy of these markers in mice with acute CVB3 myocarditis

Similar to findings in the human cohort, average myoglobin levels were higher in mice with myocarditis (1468.8 ng/mL) vs. controls (1032.3 ng/L; *p* = 0.02). There was no difference in average TnT-hs levels between mice with myocarditis vs. controls (3.05 ng/L vs 3.31 ng/L; *p* = 0.76, Fig. 4). There was moderate correlation between blood levels of TnT-hs and myoglobin (r_p_ = 0.48, R^2^ = 0.41, *p* = 0.007, Fig. 5). Similar to the human cohort, we performed ROC analysis in data obtained from mice to determine accuracy of myoglobin and TnT-hs for detecting acute myocarditis. Using the cutoff for myoglobin of 1139.2 ng/mL resulted in 70% accuracy for the detection of myocarditis, AUC = 0.68, 65% sensitivity, and 80% specificity (Fig. 6a). For TnT-hs, ROC analysis suggested an optimal cutoff value of 2.0 ng/L resulting in 43% accuracy, AUC = 0.68, 50% sensitivity, and 30% specificity.

## Discussion

This is the first study to demonstrate a strong correlation of myoglobin with LGE on CMR in patients with acute myocarditis and to suggest a robust cutoff value for myoglobin to predict LGE on CMR. The data are biologically plausible, as circulating cardiac markers such as myoglobin are frequently released during acute injury of cardiomyocytes [28, 29]. While myoglobin and CK had been used to diagnose acute coronary syndrome (ACS) in the past, cardiac troponin is now the standard of care during the last decade due its higher specificity [30, 31]. As in ACS, troponin is currently considered to be the most sensitive biomarker to detect myocarditis in those with non-obstructive coronary artery disease [32]. However, normal troponin levels do not exclude myocarditis [1, 6].

Myoglobin is an oxygen binding protein, which is typically released within 3 h of myocardial injury [33–36] followed by an elimination half-time of 5.2 min [37]. A study in myoglobin-knockout mice suggested that myoglobin plays an important role in oxygen delivery to mitochondria [38]. Horike and colleagues analyzed the degree of myoglobin staining in EMBs of patients with myocarditis using immunohistochemistry and found that myoglobin staining relates to disease severity as well as the duration of acute myocarditis [39]. Their findings are in agreement with our data suggesting higher levels of myoglobin are associated with worse disease. Since myocarditis typically presents as a systemic illness that may also affect skeletal muscle [40, 41], it is plausible that more severe acute myocarditis may be associated with the release of myoglobin from skeletal muscle, which could contribute to higher blood levels of the marker.

Our data suggest that myoglobin strongly correlates with LGE on CMR adding value as a laboratory marker to estimate disease extent. A cutoff value of 87 μg/L for myoglobin performed with 92% sensitivity, 80% specificity, and 85% accuracy in identifying patients with myocarditis and LGE on CMR.

While sensitivity and specificity of myoglobin for myocarditis appears to be superior to TnT-hs in the setting of myocarditis, studies that examined other forms of cardiac injury, e.g. in dilated cardiomyopathy and myocardial infarction, have identified TnT-hs to be the marker with the highest specificity and sensitivity, whereas myoglobin performed weakly [42, 43].

These data suggest that myoglobin is not only a valuable qualitative, but also quantitative marker for LGE in patients with myocarditis. As LGE was recently identified as a strong predictor of adverse cardiovascular outcomes [15–17], our findings suggest that myoglobin may aid in assessing risk when CMR is not immediately accessible. In line with these findings, patients with myocarditis and MACE were found to have significantly higher myoglobin levels compared to controls. A time course analysis of cardiac and inflammatory markers in myocarditis revealed peak of myoglobin levels upon admission and normalization 3 h later (Fig. 3). Therefore, it is critical that myoglobin serum levels are determined early in patients with suspected myocarditis. Myoglobin is easily accessible for analysis as a routine blood test and is available in most clinical settings at low cost (approximately 30 Euros or 58 USD/sample). Furthermore, the ability to obtain results within 1 h makes myoglobin an excellent screening tool for myocarditis. This is clinically relevant, as myocarditis continues to be underdiagnosed due to an often limited accessibility to CMR at some sites and a high threshold for EMB given its risk for complications [7]. Early recognition of myocarditis is critical for accurate diagnosis and appropriate treatment and to improve our understanding of the disease. Surprisingly, correlation of TnT-hs and CK with LGE were only weak for patients with myocarditis in this study, but aligns with a recently published study analyzing the correlation of LGE with TnT-hs and CK, but not with myoglobin in patients with suspected myocarditis [44].

Importantly, we tested the newly developed cutoff for serum myoglobin with TnT-hs, the current laboratory gold standard for the diagnosis of myocarditis. First, we applied ROC analysis to investigate an optimal cutoff for TnT-hs to identify patients with myocarditis and LGE. Using an optimized cutoff of 201 ng/L, diagnostic sensitivity of TnT-hs was 77% with only 48% specificity, and AUC = 0.60. Without prior optimization of the cutoff and applying the standard reference range of TnT-hs [0–14 ng/L], specificity decreased further to 22%.

Finally, we reproduced our findings in a clinically translational mouse model of CVB3 myocarditis that uses heart-passaged virus to induce disease. While elevated myoglobin levels detected myocarditis in mice with 70% accuracy, diagnostic accuracy for TnT-hs was only 43%.

In summary, our data suggest that serum myoglobin is a valuable screening tool to identify patients with myocarditis and LGE on CMR as it peaks early, reflects acuity due to its short lifespan, and appears to perform more accurately in acute myocarditis than the current laboratory gold standard TnT-hs. The data are biologically plausible, as myoglobin is released during cardiac injury [28, 29, 45] and its expression on EMBs increases with the severity of myocarditis [39]. Our findings on myoglobin obtained from the human myocarditis cohort reproduced in a clinically relevant mouse model of CVB3 myocarditis. Our data further suggest that myoglobin provides qualitative, quantitative, and prognostic information with strong correlation to LGE—a risk predictor of MACE. Availability of serum myoglobin as a routine blood test in most clinical settings for low cost and the ability to obtain results within 1 h make it a potentially valuable diagnostic tool for broad screening and clinical decision making to triage patients for CMR.

We show for the first time that serum myoglobin strongly correlates with LGE on CMR in acute myocarditis and that myoglobin may serve as a surrogate for severe myocardial damage in patients with myocarditis. Since myoglobin is available in most emergency rooms as a routine laboratory test, it may add value in guiding triage for CMR. This could be particularly helpful in hospitals without a CMR infrastructure where patients are transferred to a tertiary care center for further imaging.

## Electronic Supplementary Material

ESM 1 (DOCX 249 kb)

## Abbreviations

aHTN: Arterial hypertension
CAD: Coronary artery disease
CMR: Cardiac magnetic resonance imaging
DCM: Dilated cardiomyopathy
DM: Diabetes mellitus
EMB: Endomyocardial biopsy
CK: Creatine kinase
CRP: C-reactive protein
CRT-P: Cardiac resynchronization therapy pacemaker
ICD: Implantable cardioverter defibrillator
Lc: Leukocytes
LGE: Late gadolinium enhancement
LVEF: Left ventricular ejection fraction
NT-pro BNP: NT-pro brain natriuretic peptide
PVC: Premature ventricular contractions
r_s_: Spearman rank correlation coefficient
SD: standard deviation
Tc: Thrombocytes
TnT-hs: High sensitivity troponin T
VT: Ventricular tachycardia
