# Supplementary material for: Myoglobin for Detection of High-Risk Patients with Acute Myocarditis
Source: J Cardiovasc Transl Res. 2020 Jan 31;13(5):853–63. doi: 10.1007/s12265-020-09957-8 (PMC7541375; doi:10.1007/s12265-020-09957-8)
Supplement: Supplementary file 1 — (DOCX 249 kb) [file 12265_2020_9957_MOESM1_ESM.docx]

**SUPPLEMENTAL DATA**

**Methods**

**CMR Imaging**

LGE short axis images were generated 10 minutes after intravenous administration of a gadolinium-based contrast agent. Routine CMR reporting included evaluation of left ventricular ejection fraction (LVEF) and wall motion abnormalities. Pericardial involvement was defined as pericardial thickening or effusion on CMR. Two experienced analysts of CMR interpreted all imaging data for this study under the oversight of the director of cardiac imaging in our division. Data analysis was performed in a blinded manner. Images were post-processed using the software GT Volume from GyroTools LCC ® for planimetry. A region of interest was manually drawn around the area of LGE, delineated as bright areas in each slice in short-axis view. To include only myocardial LGE, endocardium and epicardium were manually delineated (supplemental data, Figure S1). LGE extent was calculated as percentage of left ventricular myocardial volume. Intraobserver agreement was tested in a subgroup of 10 patients, and interobserver agreement between the two analysts was tested in a subgroup of 5 patients by linear regression and Bland-Altman analysis similar to prior studies ([24](#_ENREF_24),[46](#_ENREF_46),[47](#_ENREF_47)). A p value > 0.05 was considered not significant.

**Statistical analysis**

In order to determine optimal cutoff serum levels of the newly developed biomarker and TnT-hs to detect LGE in myocarditis, ROC analysis was applied to the primary cohort. Cutoff values obtained from ROC analysis as well as the standard reference range of TnT-hs were validated in an independent cohort of patients with myocarditis and controls to assess diagnostic accuracy of these makers. All statistical analyses were performed with SPSS (version 23 SPSS, Chicago, IL, USA) and Stata (version Stata/SE 13.1, Texas, USA). The first author had full access to all data in the study and takes responsibility for its integrity and the data analysis.

**SUPPLEMENTAL FIGURES**

**Figure S1**


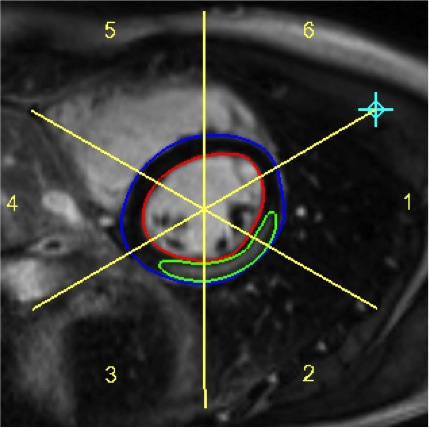


**SUPPLEMENTAL TABLES**

**Table S1.** Detailed LGE measurements in individual patients with myocarditis (n = 44)

| Individual  Patients | Total LGE (mL) | Total left ventricular myocardial volume (mL) | LGE% of left ventricular myocardial volume |
| --- | --- | --- | --- |
| 1  2  3  4  5  6  7  8  9  10  11  12  13  14  15  16  17  18  19  20  21  22  23  24  25  26  27  28  29  30  31  32  33  34  35  36  37  38  39  40  41  42  43  44 | 1.20  2.06  4.34  3.75  20.52  5.52  5.37  5.16  4.93  4.04  25.74  35.86  14.56  49.17  10.66  10.35  7.33  5.94  4.00  49.13  14.63  20.96  9.30  13.31  8.07  35.54  20.87  4.29  7.29  20.34  6.09  20.70  6.47  5.63  52.72  9.54  10.44  3.10  5.31  3.22  1.32  1.62  2.54  2.91 | 82.22  110.10  124.62  169.32  39.93  82.77  113.03  115.31  159.74  140.98  134.38  195.57  110.19  174.03  89.98  92.12  109.87  130.79  123.78  145.81  161.04  222.48  133.52  144.13  151.89  134.48  129.70  90.16  102.27  169.16  102.14  89.30  106.20  95.64  227.46  164.98  117.72  124.27  152.73  80.13  66.75  57.80  102.47  90.85 | 1.46  1.87  3.48  2.21  14.66  6.67  4.75  4.47  3.09  2.87  19.15  18.34  13.21  28.25  11.85  11.24  6.67  4.54  3.23  33.69  9.08  9.42  6.97  9.23  5.31  26.43  16.05  4.76  6.86  12.02  5.96  23.18  6.09  5.89  23.18  5.78  8.87  2.49  3.48  4.02  1.98  2.80  2.48  3.20 |
